# Tasmanian devils with contagious cancer exhibit a constricted T-cell repertoire diversity

**DOI:** 10.1038/s42003-019-0342-5

**Published:** 2019-03-13

**Authors:** Yuanyuan Cheng, Mariano Makara, Emma Peel, Samantha Fox, Anthony T. Papenfuss, Katherine Belov

**Affiliations:** 1 UQ Genomics Initiative, The University of Queensland, St. Lucia, QLD 4072 Australia; 2 School of Life and Environmental Sciences, The University of Sydney, Sydney, NSW 2006 Australia; 3 Department of Primary Industries, Parks, Water and Environment, 134 Macquarie Street, Hobart, Tasmania 7000 Australia; 4 Bioinformatics Division, The Walter and Eliza Hall Institute of Medical Research, Parkville, VIC 3052 Australia; 5 Computational Cancer Biology Program, Peter MacCallum Cancer Centre, Melbourne, VIC 3000 Australia; 6 Department of Medical Biology, University of Melbourne, Melbourne, VIC 3010 Australia; 7 Sir Peter MacCallum Department of Oncology, University of Melbourne, Melbourne, VIC 3010 Australia

## Abstract

The Tasmanian devil (*Sarcophilus harrisii*) is threatened by a contagious cancer, known as Devil Facial Tumour Disease (DFTD). A highly diverse T-cell receptor (TCR) repertoire is crucial for successful host defence against cancers. By investigating TCR beta chain diversity in devils of different ages, we show that the T-cell repertoire in devils constricts in their second year of life, which may explain the higher DFTD prevalence in older devils. Unexpectedly, we also observed a pronounced decline in TCR diversity and T cell clonal expansion in devils after DFTD infection. These findings overturned the previous assumption that DFTD did not directly impact host immunity.

## Abstract (web summary)

Yuanyuan Cheng et al. showed that the T-cell repertoire diversity of Tasmanian devils diminishes during their second year of life which may explain the prevalence of devil facial tumor disease in older devils. Infection with this disease also impacts T-cell diversity highlighting a previously unknown effect of the devil facial tumor disease on host immunity.

## Introduction

The Tasmanian devil (*Sarcophilus harrisii*, referred to as the devil hereinafter), the world’s largest living carnivorous marsupial, is currently at risk of extinction in the wild due to an unusual transmissible cancer known as Devil Facial Tumour Disease (DFTD)^1^. DFTD is caused by a clonal tumour cell line that originated in a Schwann cell^2^. Tumour cells are transmitted between individuals as allografts via biting^3^, and diseased animals usually die within 6 months^4^. Despite having a typical mammalian immune system which is capable of allograft rejection^5–7^, most devils produce no allogeneic response against the foreign tumour cells, with naturally occurring anti-DFTD responses having only been observed in six animals so far^8^. Such lack of immune response has thus far been explained by the fact that DFTD cells downregulate their cell surface expression of the major histocompatibility complex (MHC) class I molecules through epigenetic regulation^9^. However, MHC I downregulation is one of the most common mechanisms of tumour immune escape in human cancers^10,11^, yet there has been no indication that such cancers can cross histo-incompatibility barriers. Therefore, it is likely that DFTD uses additional immune evasion mechanisms to be so remarkably contagious.

The hypothesis that we explored in this study arose from the observation that DFTD does not affect devils of different age classes equally. Devils have an average lifespan of 5–6 years. Females become reproductively mature at the age of 1 and males at the age of 2 years^12^. At DFTD-affected sites, devils older than 3 years of age are rare (<10%, with certain populations containing only animals <3 years of age), and the level of disease prevalence is higher in older devils than that in younger animals^13^. Interestingly, animals less than 1-year-old are rarely affected by DFTD^1^, and diseased mothers do not transmit DFTD to their young^14^. Such age-associated difference in disease prevalence led us to hypothesise that the decline of overall immune competence in an individual, either due to ageing or other factors, could play a role in causing susceptibility to DFTD.

To perform a comprehensive characterisation of immunosenescence in the devil and explore its role in DFTD, we analysed the T-cell repertoire diversity of devils. T cells are the main effector cells responsible for the immune surveillance of infections and tumours. T-cell repertoire diversity, which decreases with age as immunosenescence advances, is a key indicator of the capacity of an animal’s immune system to respond to diseases^15,16^. We focused on the T-cell receptor (TCR) beta (TCRB) chain, which is believed to harbour most of the diversity within the T-cell repertoire, particularly in its third complementarity-determining region (CDR3), which is the main region involved in antigen recognition^17^. In humans, it has been estimated that the number of unique TCRB sequences can exceed 1 × 10^8^ in a young adult^18^. This extraordinary diversity is generated during maturation of naive T cells in the thymus via a mechanism known as VDJ recombination, which involves random recruitment and imprecise joining of TCR variable (V), diversity (D) and joining (J) gene segments^19^. Having a highly diverse repertoire of T cells is not only essential for successful host defence against a wide range of pathogens, but also has important implications for an animal’s survival in the face of cancer. For instance, studies in humans have shown that high TCRB diversity and less clonotype loss during immunotherapy are associated with better clinical outcomes and improved overall survival in patients with metastatic melanoma^20,21^. In light of this, we set out to investigate the role of TCRB diversity in DFTD. We observed a marked decline of TCRB diversity in devils within their first 2 years of life, resulting in early immunosenescence and increased susceptibility of older animals to DFTD. Strikingly, we also found that devils’ T-cell repertoire undergoes further constriction after DFTD infection, rendering the animals more vulnerable to other secondary infections and cancers.

## Results

### TCRB transcript sequencing

**Fig. 1:**
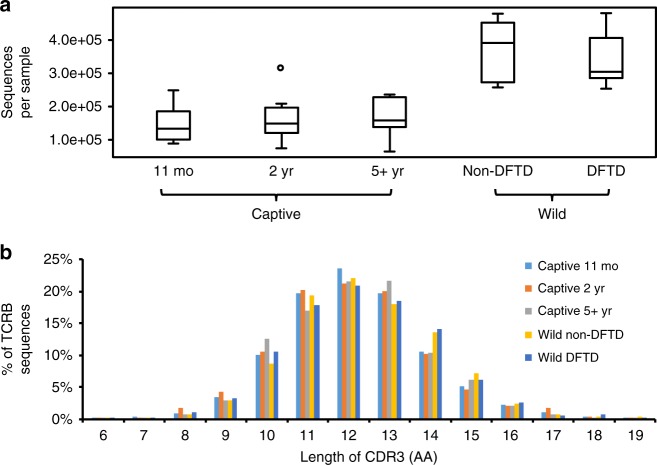
Characterisation of the TCRB sequence dataset. **a** The number of sequences per sample; captive and wild samples were analysed separately due to the differences in sequencing depth. Box-plot elements: centre line, median; box limits, first and third quartiles; whiskers, minimum and maximum ranges; points, outliers. **b** Length distribution of the CDR3 region

We analysed peripheral blood of 50 devils, including 32 DFTD-free captive devils at 11 months (*N* = 10), 2 years (*N* = 10) or more than 5 years (*N* = 12) of age, and -2 or 3-year-old wild devils with (*N* = 7) or without (*N* = 8) DFTD; additionally, paired samples were collected from three devils before and after catching DFTD (Supplementary Table 1). TCRB partial transcripts consisting of a partial V segment, D, J and 5′ end of C were amplified and sequenced on an Illumina Miseq System (NCBI SRA# SRP092288). The final dataset comprises a total of 12,517,846 quality-filtered sequences, with the number of sequences per sample ranging between 64,822 and 480,643 (Fig. 1a, Supplementary Table 1). From these sequences, 1,749,761 distinct TCRB transcripts representing distinct TCR clonotypes were identified by clustering with CD-HIT. Consistent with human and mouse genes, devil TRBV segments contain a conserved cysteine residue near the 3′ end and TRBJ segments contain a conserved phenylalanine in the 5′ portion, which are used to define the beginning and ending of CDR3 in TCRB transcripts^22^. More than 84% of devil CDR3 sequences had a length between 30 and 42 bp (Fig. 1b), similar to the length distribution in humans^23^. It should be noted that this study aims to compare TCR diversity between sample groups; due to the exceptionally high diversity of the T-cell repertoire, deeper sequencing will be necessary to fully reveal the diversity in devils.

### Early immunosenescence in the devil

**Fig. 2:**
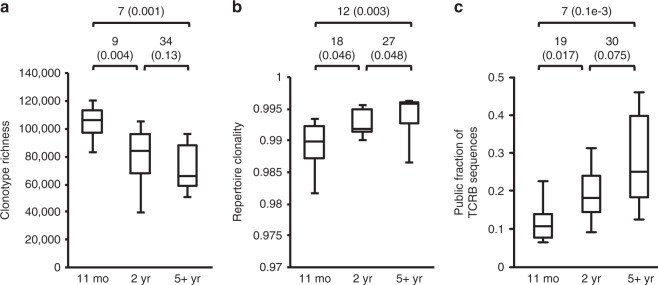
Comparison of TCRB diversity among three age groups using captive devils. Mann–Whitney U-test statistics and *p*-values (in parentheses) are shown above each panel. Box-plot elements: centre line, median; box limits, first and third quartiles; whiskers, minimum and maximum ranges. **a** TCRB clonotype richness estimated using DivE. **b** Clonality of the TCR repertoire inferred by Gini coefficient. **c** Proportion of sequences that represent public clonotypes

Captive devils were used to study the pattern of immunosenescence in the species. The diversity of the TCRB repertoire was estimated using the Chao1^24^, Shannon entropy^25^ and Gini coefficient^26^, which are commonly used in ecological studies, but can also be applied to immunological data^17^. A more recently developed method, DivE, was also used, as it has been proposed to produce a more accurate estimation than other methods for datasets with relatively low sequencing depth^27^. To account for the differences in sequencing depth among samples, all captive devil samples were rarefied (repeated random subsampling) to a common size of 100,000 sequences, while wild samples were rarefied to 250,000 sequences per sample. The different indices produced consistent results (Fig. 2, Supplementary Figure 1).

Devils aged 11 months had the most diverse TCRB repertoire and showed the highest richness and evenness (Fig. 2a, b). Compared with the young devils, both 2-year-old and 5-year-old adult devils had significantly lower levels of TCRB diversity (Fig. 2a, b), showing a clear trend in the decline in richness and increase in clonality with age, although no significant difference was detected between 2-year-old and older devils in repertoire richness. The relative abundance of public clonotypes (identical clonotypes shared by multiple individuals, as opposed to private clonotypes unique to an individual) also increased significantly between young and adult devils (Fig. 2c), which likely resulted from decreased output of new T-cell clonotypes by the thymus and accumulation of clonal selection caused by common pathogens affecting devils^28^.

**Fig. 3:**
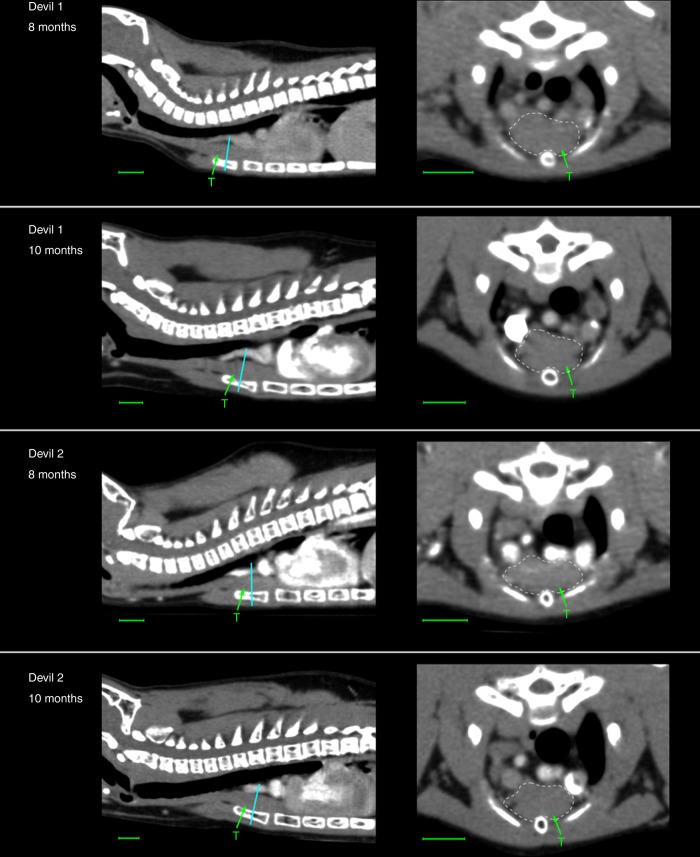
Computed tomography imaging of the thymus (T) in young devils. Cyan lines indicate locations of transverse sections where measurements of the thymus shown in Table 1 were taken. Scale bar under each image represents 1.00 cm

These observations are indicative of an early onset of immunosenescence in the devil, causing a major decline in the T-cell repertoire diversity prior to the age of 2. We further investigated this by examining the thymus of young devils via computed tomography (CT) imaging. The thymus is the sole organ responsible for the production of naive T cells and novel clonotypes, and plays a key role in generating the high TCR diversity during development. The thymus is known to undergo structural changes and shrinks in total mass with age^29^. This degeneration process, known as thymic involution, represents the most drastic anatomical change of the immune system in relation to ageing. One male and one female devil were scanned at two time points: 8 months and 10 months of age. CT images revealed that the thymus of devils is situated in the typical mammalian position, dorsal to the sternum, ventral to the great vessels and the trachea and cranial to the heart (Fig. 3). A cervical prolongation is also indicated in the CT images, extending some distance cranially along the trachea. Based on estimation of the cross-sectional area of the thickest part of the gland, enlargement of the thymus in devils is likely completed before the age of 8 months, with marginal changes in size (0.3% in the female and −0.7% in the male) occurring between the two time points studied (Table 1). Signs of involution can be seen in the thymus at 10 months of age, with the outer layer of cortical tissue starting to degenerate and showing disruption (Fig. 3 transverse sections).

**Table 1:** Measurements of the thymus in young devils

	Sex	Age	Body mass (kg)	Thymus cross-sectional area (mm^2^)
Devil 1	Female	8 months	1.17	86.6
		10 months	1.30	86.9
Devil 2	Male	8 months	1.81	88.7
		10 months	2.10	88.1

### DFTD-associated constriction of TCRB diversity

**Fig. 4:**
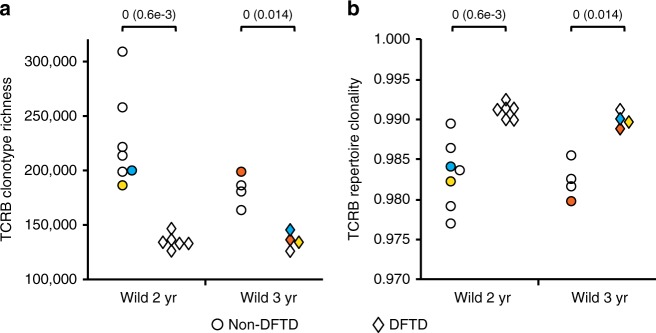
Comparison of TCRB diversity between DFTD and non-DFTD devils. Mann–Whitney U-test statistics and *p*-values (in parentheses) are shown above each panel. Coloured data points represent three pairs of pre- and post-DFTD samples, with the same colour indicating the same animal. **a** TCRB clonotype richness estimated using DivE. **b** Clonality of TCR repertoire inferred by Gini coefficient

Analysis of the T-cell repertoire diversity in wild devils revealed a striking difference between DFTD-affected and unaffected individuals. As shown in Fig. 4, diseased devils at 2 or 3 years of age had a T-cell repertoire that was considerably less diverse than that of non-DFTD animals, with the average richness decreasing by 40.4 and 25.2% in diseased 2-year-old and 3-year-old devils, respectively, compared with non-DFTD devils. This loss of TCR diversity in DFTD-affected devils was characterised by a reduced number of distinct TCRB clonotypes and a decreased level of evenness, which are indicative of T-cell repertoire constriction and clonal expansion occurring in association with DFTD. Furthermore, comparisons of TCR diversity in three devils before and after catching DFTD showed a consistent pattern with the diversity dropping to a level markedly lower than that seen in non-DFTD devils of the same age (Fig. 4), suggesting that such constricted TCR repertoire was unlikely a consequence of ageing or a pre-existing condition of the devils before catching DFTD.

**Fig. 5:**
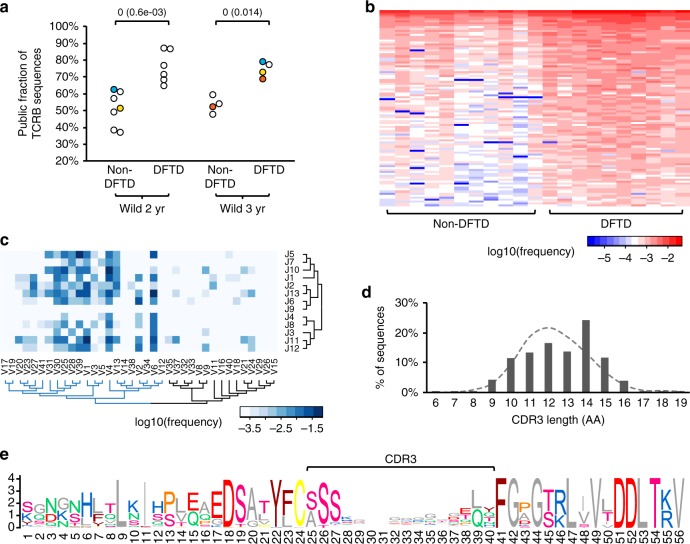
Expansion of public clonotypes in DFTD-affected devils. **a** Prevalence of public clonotypes significantly increased in diseased devils. Mann–Whitney U-test statistics and *p*-values (in parentheses) are shown. Coloured data points represent three pairs of pre- and post-DFTD samples, with the same colour indicating the same animal. **b** Observed frequencies of top-100 most abundant public clonotypes that showed significantly higher frequencies (Mann–Whitney U test cutoff *p* < 0.05) in DFTD samples than in non-DFTD samples. **c** TRBV and TRBJ segment usage among expanded public clonotypes. **d** Expanded TCRB sequences tend to have a long CDR3 domain compared with the average distribution of CDR3 length across all sequences (dashed line). **e** Sequence logo showing a high variability within the CDR3 domain in expanded clonotypes

Further dissection of data revealed a pronounced change in the relative abundance of public TCRB clonotypes between non-DFTD and DFTD devils, with the T-cell repertoire of diseased animals becoming dominated by public clonotypes (Fig. 5a). Among 85,632 public clonotypes identified, 4889 showed significant increase in observed frequencies in the DFTD group (Mann–Whitney U test *p*-value < 0.05) and were highly shared across diseased devils (Fig. 5b; observed frequencies and nucleotide sequences of the top-100 most abundant of these clonotypes are available in Supplementary Data 1–2). Out of 451 detected V and J segment combinations (Supplementary Figure 2), 183 were found in these 4889 clonotypes, with most of the utilised V segments belonging to the same phylogenetic clade (Fig. 5c). Although no pattern was observed in CDR3 sequences across these clonotypes, they appeared to tend to have a relatively long CDR3 domain (Fig. 5d, e). These results indicate that the constricted TCR diversity in DFTD devils was largely caused by selective clonal expansion in the T-cell repertoire, resulting in a large fraction of shared clonotypes amongst individuals.

**Fig. 6:**
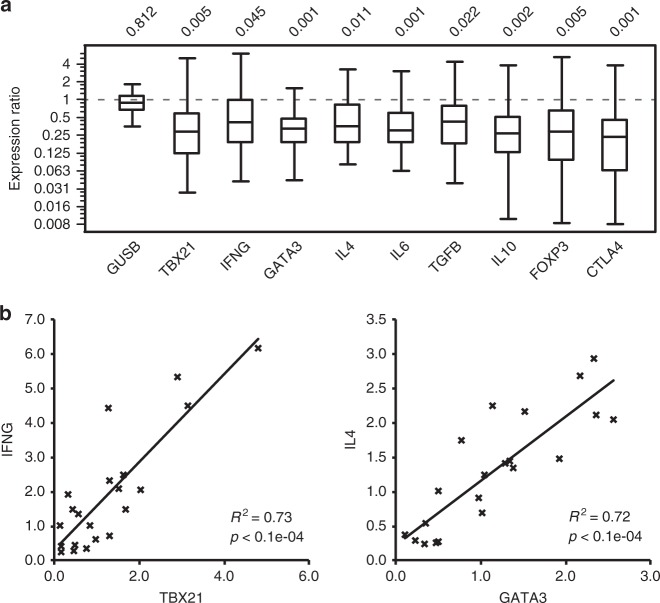
Relative quantitative PCR analysis of gene expression in DFTD and non-DFTD devils. **a** Fold change in the expression level of nine T-cell marker, transcription factor or cytokine genes in DFTD devils in comparison with non-DFTD devils (log-2 scale). Box-plot elements: centre line, median; box limits, first and third quartiles; whiskers, minimum and maximum ranges. **b** Association between T-bet and IFN-γ gene expression, and between GATA-3 and IL-4 expression in wild devils. Gene expression was normalised to two reference genes, *GAPDH* and *GUSB*. Relative expression of *GUSB* normalised to *GAPDH* is shown (in panel **a**) as a control for non-differentiated expression between DFTD and non-DFTD samples

As DFTD can be accompanied by secondary infections^30^, further analysis was carried out to examine if the observed DFTD-associated clonal expansion within the TCR repertoire could have resulted from responses to secondary infections. Quantitative PCR was carried out to assess the peripheral blood level of nine T-cell-related markers, transcription factors and cytokines in DFTD-affected devils relative to that in non-DFTD devils (Fig. 6a). Cytokines interferon gamma (encoded by *IFNG*), interleukin 4 (*IL4*) and interleukin 6 (*IL6*), which are centrally involved in differentiation and robust effector responses of different subsets of T cells, such as Th1, Th2 and Th17^31–35^, all showed decreased expression levels in diseased devils in comparison with the baseline expression in non-DFTD devils. The expression of IFN-γ and IL-4 was closely associated with that of transcription factors T-bet (*TBX21*) and GATA-3 (*GATA3*), respectively, which were also suppressed in the DFTD group (Fig. 6b). These patterns are inconsistent with a cellular or humoral immune response to an infectious disease, suggesting that the observed decline in TCR diversity in DFTD-affected devils was unlikely the result of secondary infections. We also explored the possibility of regulatory T cells causing immune suppression by examining the expression of transforming growth factor beta 1 (*TGFB1*), interleukin 10 (*IL10*), forkhead box P3 (*FOXP3*) and cytotoxic T-lymphocyte protein 4 (*CTLA4*), all of which are known to positively correlate with the population size of immunosuppressive CD4+CD25+ Treg cells^36,37^. The expression of these genes was also downregulated in the DFTD group (Fig. 6a), suggesting that the prevalence of Treg cells in the peripheral blood of DFTD-affected devils was not elevated but suppressed.

## Discussion

In this study, we found a negative correlation between age and TCRB diversity in devils, and a marked decrease in TCRB diversity associated with DFTD infection. Our results suggest that devils experience a depletion in TCRB repertoire diversity between the age of 11 months and 2 years, possibly due to an early onset of thymic involution. One important feature of immunosenescence in the devil is that the rate of the T-cell diversity decline during early life is much higher than what is observed in the human. In humans, the change in TCRB diversity between childhood and young adulthood (between 6 and 25 years) is not prominent^38^. Also, interestingly, in humans, the TCRB diversity declines substantially between the ages of 30s and 60s and then remains relatively stable afterwards^38^, whereas in devils, the decline in clonotype richness between 2-year-olds and 5-year-olds was not statistically significant. These findings may indicate that immunosenescence advances at a faster rate in early life in the devil, with the thymus becoming mostly regressed before adulthood and therefore ceasing to produce new naive T cells; older devils possibly mainly rely on peripheral T-cell division to maintain the T-cell pool, as further indicated by the observed increase in repertoire clonality with age.

Limited literature is available on the thymic development and involution in the devil and other closely related species. Marsupials have a distinctive morphology and life history compared with eutherian mammals. Due to the short gestation periods (15–35 days), marsupial neonates are highly underdeveloped and lack lymphoid tissue and lymphocytes at birth^39^. However, this is compensated by rapid development of immune tissue immediately after birth, with the thymus and lymphocytes appearing within the first week^39^. In koalas (*Phascolarctos cinereus*), thymic development continues until 8 months of age, and involution does not start until 2–3 years after sexual maturity^40^. In the devil, however, we observed in the two examined individuals that the thymus had stopped enlargement at 8 months and showed signs of involution at 10 months. This is consistent with a previous histological and immunohistochemical study finding that the thymus had started to regress in 1-year-old devils^5^. These observations indicate that the onset of thymic involution in the devil likely occurs in the first few months of life, long before puberty starts, in contradiction to koalas. This contradiction may be due to the very different life histories of the two species; it may also be related to the fact that koalas, like other diprotodont marsupials^39^, such as kangaroos and wallabies, have a different thymic system comprising one thoracic and one cervical thymus, which may undergo different development and atrophy phases than the common single-thymus system found in devils. Further work on devil pouch young will be required to determine the exact timeline of thymic development and involution in the devil.

Before this study, it was assumed that DFTD infection did not have a direct impact on the normal functionality of the host immune system. This assumption was based on in vitro experiments, which demonstrated that lymphocytes isolated from diseased devils were able to produce comparable levels of proliferation responses to mitogen stimulation as those from healthy individuals^41^. Our results overturned this assumption, showing that DFTD caused substantial constriction in the TCRB repertoire of affected animals. It has been seen in mice that, despite having functional CD8 T cells, aged mice lose the capability to mount CD8 T-cell responses to certain influenza virus epitopes due to restricted TCRB diversity^16^. The same process may be occurring in diseased devils, with the depleted diversity in the TCRB repertoire of DFTD-affected devils directly impairing immunity to infections. Moreover, based on qPCR analysis, a range of cytokines that play crucial roles in T-cell differentiation, homoeostasis and activation were produced at lower levels in the peripheral blood of DFTD-affected devils. These results suggest that DFTD not only hides from the host immune surveillance by downregulating MHC molecules on tumour cells^9^, but can also exert a negative impact on the host immune system, which in turn can facilitate its transmission.

The decreased TCR diversity in diseased devils was largely due to the relative expansion of certain clonotypes, which tended to have a long CDR3 domain and utilise certain sets of V segments. These selectively expanded clonotypes that were shared and dominant among DFTD-affected individuals were inferably ineffective against the tumour and had lower cytokine production. The ultimate mechanism underlying this clonal selection remains to be investigated; one possible hypothesis is that in young devils, thymic production of new naive T cells may enable a continual generation of novel clonotypes that have a higher affinity towards DFTD tumour antigens, which may be a prerequisite for maintaining a tuned T-cell population to defend against the tumour.

A range of human diseases have been reported to cause constriction of TCR diversity, such as HIV^42^, bladder cancer^43^ and glioma^44^, via mechanisms such as T-cell apoptosis and induced thymic involution. Therapeutic treatments, such as chemotherapy or radiation, are also known to lead to a depleted diversity of the T-cell repertoire^45,46^. By contrast, certain cancer immunotherapy, such as CTLA4 blockade, can induce T-cell activation and proliferation, and increase the number of TCR clonotypes and diversity^20^. This highlights the importance of further studies to explore the mechanism of DFTD-induced TCRB repertoire disruption for the development of an efficient treatment. It will also be important to characterise how the disease impacts on γδ T cells and other lymphocyte populations of the host, which will provide comprehensive insights into this unusual transmissible cancer.

To sum up, the constriction of TCRB repertoire in the devil’s first 2 years of life likely has contributed to the observed higher DFTD prevalence in older devils. DFTD infection can lead to further decline of the TCRB diversity in diseased animals and result in a repertoire dominated by certain clonotypes with high reoccurrence among individuals. These results have important implications for the ongoing development of anti-DFTD vaccines^47^ and treatments^48^. Our data suggest that animal’s age needs to be a key consideration during vaccination, as the constricted TCR repertoire of older devils is likely to cause poor responses to vaccination. Further research is needed to elucidate the mechanism underlying DFTD-induced T-cell repertoire disruption, which may lead to novel treatment strategies and improved success rates of therapies.

## Methods

### Annotation of devil TCRB genes

Three TRBC and 41 V segments were identified in the devil reference genome using multiple rounds of BLAST and HMMER searches (detailed in Supplementary Methods). A schematic map of the devil TCRB locus is shown in Supplementary Figure 3, with coordinates of all annotated TCRB gene segments provided in Supplementary Table 2.

### Sample collection

Peripheral blood was collected (Supplementary Methods) from 32 captive devils, which belonged to three distinct age groups: 11 months old (*N* = 10), 2 years old (i.e. 24–36 months after birth; *N* = 10) and more than 5 years old (*N* = 12). Fifteen 2–3-year-old wild devils with (*N* = 7) or without (*N* = 8) DFTD were sampled, with three additional pairs of samples collected from three devils before and after catching DFTD (Supplementary Table 1). These procedures were carried out with approval from the Animal Ethics Committee of The University of Sydney under project number 550 (captive devils) and 681 (wild devils).

### TCRB transcript amplification and sequencing

The devil blood RNA was extracted using RNeasy Protect Animal Blood Kit (Qiagen) with on-column DNase treatment. Quality and concentration of extracted RNA was analysed on a 2100 Bioanalyzer (Agilent Technologies). All samples had an RNA integrity number higher than 8.6. Blood cell counts were performed on a Sysmex XT-2000iV Haematology Analyzer. Similar to what was found in humans^49^, the blood RNA level positively correlates with white blood cell count (R^2^ = 0.33) and lymphocyte count (R^2^ = 0.75) in devils (Supplementary Figure 4). In total, 500 ng of RNA was used in cDNA synthesis with SuperScript VILO Master Mix (Invitrogen). The strategy for TCRB transcript amplification was adapted from a protocol developed in humans^18^. Instead of targeting full-length TCRB transcripts, this strategy amplifies shorter partial transcripts, which allows for an overlap between Illumina paired-end reads over the CDR3 region and thereby ensures high consensus sequence accuracy. This method is more suitable for comparative analysis of TCR diversity among samples^18^, but can be less accurate for evaluating expression levels of various V segments within a sample due to the differences in PCR primer efficiencies. Briefly, TCRB transcripts were amplified using forward primers specific to one to three V segments and a universal reverse primer designed for all three C segments; primers were tagged with Illumina adaptor sequences on the 5′ end and designed and optimised to have high efficiencies (>0.95; Supplementary Table 3). Successful amplification was confirmed by visualising PCR amplicons on gels. Four TRBV segments (V7, V10, V22 and V36) consistently showed no amplification among samples with multiple primers designed and tested, and therefore are likely not transcribed. PCR was carried out using Platinum Taq DNA polymerase high-fidelity kit (Invitrogen, protocol provided in Supplementary Methods). PCR products were pooled for each sample and amplicons with 250–350 bp of size were purified from the gel using QIAquick Gel Extraction Kit (Qiagen). Purified amplicons were submitted to The Ramaciotti Centre for Genomics (Randwick, Australia) for library preparation service and sequencing on an Illumina Miseq in two 2 × 200-bp paired-end runs.

### Sequence data analysis

Paired-end reads were joined using fastq-join method^50^. TCRB sequences containing a minimum Phred quality score higher than 30 (Supplementary Figure 5) and an intact open-reading frame were retained. The TRBV region of the sequences were matched to the 37 transcribed germline TRBV segments using BLAST. TRBJ sequences were isolated by trimming 5′ V and 3′ C regions, and were used to identify a total of 13 TRBJ segments in the devil genome. Sequences that failed to map to annotated V and J segments (3.1% of all sequences) were excluded from subsequent analyses. CDR3 was defined as the region between the conserved cysteine near the 3′ end of TRBV and the conserved phenylalanine in the 5′ portion of TRBJ segments^22^. Sequences were clustered and distinct TCRB transcripts were identified using the CD-HIT method^51^. Transcripts that were detected in more than one animal with 100% sequence identity were defined as public TCRB. To account for the differences in sequencing depth among samples, all captive samples were rarefied to a common size of 100,000 sequences and wild samples rarefied to 250,000 sequences with 10 rounds of subsampling performed. The level of diversity was estimated using four methods: Chao1^24^, Shannon index^25^, Gini coefficient^26^ and DivE^27^; with DivE, the curvature parameter (C_p_) values ranged between 0.20 and 0.33. Mann–Whitney U tests were performed to identify significant differences (*p* < 0.05) between sample groups. Evolutionary relationships between TRBV and TRBJ segments were inferred using the Neighbour-Joining method.

### Computed tomography

Two young devils, one male and one female, were used to investigate the development/involution of the thymus. Both animals were hand-raised, belonging to the Save the Tasmanian Devil Program. CT scans were taken at two time points, 8 months and 10 months of age (Supplementary Methods). As devils are expected to enter puberty in their second year, further scans were not performed to reduce the impact on the animals’ development. CT images were analysed and measurements of the thymus were taken using software OsiriX. This work was approved by the Animal Ethics Committee of The University of Sydney under project number 550.

### Quantitative PCR

Relative expression quantification of nine target genes, including *TBX21, IFNG*, *GATA3, IL4*, *IL6*, *TGFB1*, *IL10*, *FOXP3* and *CTLA4*, was carried out using two previously established reference genes *GAPDH*^2^ and *GUSB*^52^ on DFTD and control (non-DFTD) samples. Primers and PCR protocols for *IL10*, *IL6*, *TGFB1*, *GAPDH*, *GUSB, TBX21, IFNG*, *GATA3* and *IL4* were adopted from previous publications^2,12,52^. Primers for *FOXP3* and *CTLA4* were designed using software Oligo v6.71, with forward and reverse primers located on different exons (all primer sequences in Supplementary Table 4). Primer specificity was checked by purifying and sequencing the PCR amplicon. Real-time PCRs were carried out on a RotorGene 6000 using Quantifast Sybr Green PCR Master Mix (Qiagen; Supplementary Methods). PCR efficiencies ranged between 0.95 and 1.04, and the correlation coefficient of standard curves between 0.988 and 1.000 (Supplementary Table 4). Normalised relative expression of target genes was calculated using the geNorm equations^53^. Comparisons between DFTD and control groups were performed in software REST 2009 v2.0.13, with *p* < 0.05 suggesting down- or upregulated expression in the disease group^54^. The relative expression of the reference gene *GUSB*, normalised to *GAPDH*, was included in Fig. 6 as a control for non-differentiated expression.

### Reporting summary

Further information on experimental design is available in the Nature Research Reporting Summary linked to this article.

## Supplementary information

Supplementary Information

Description of Additional Supplementary Files

Supplementary Data 1

Supplementary Data 2

Reporting Summary

## Data Availability

Sequence data that support the findings of this study have been deposited in NCBI Sequence Read Archive (SRA) with the accession code SRP092288.
