# Supplementary material for: Tasmanian devils with contagious cancer exhibit a constricted T-cell repertoire diversity
Source: Commun Biol. 2019 Mar 13;2:99. doi: 10.1038/s42003-019-0342-5 (PMC6416256; doi:10.1038/s42003-019-0342-5)
Supplement: Supplementary file 1 — Supplementary Information [file 42003_2019_342_MOESM1_ESM.pdf]

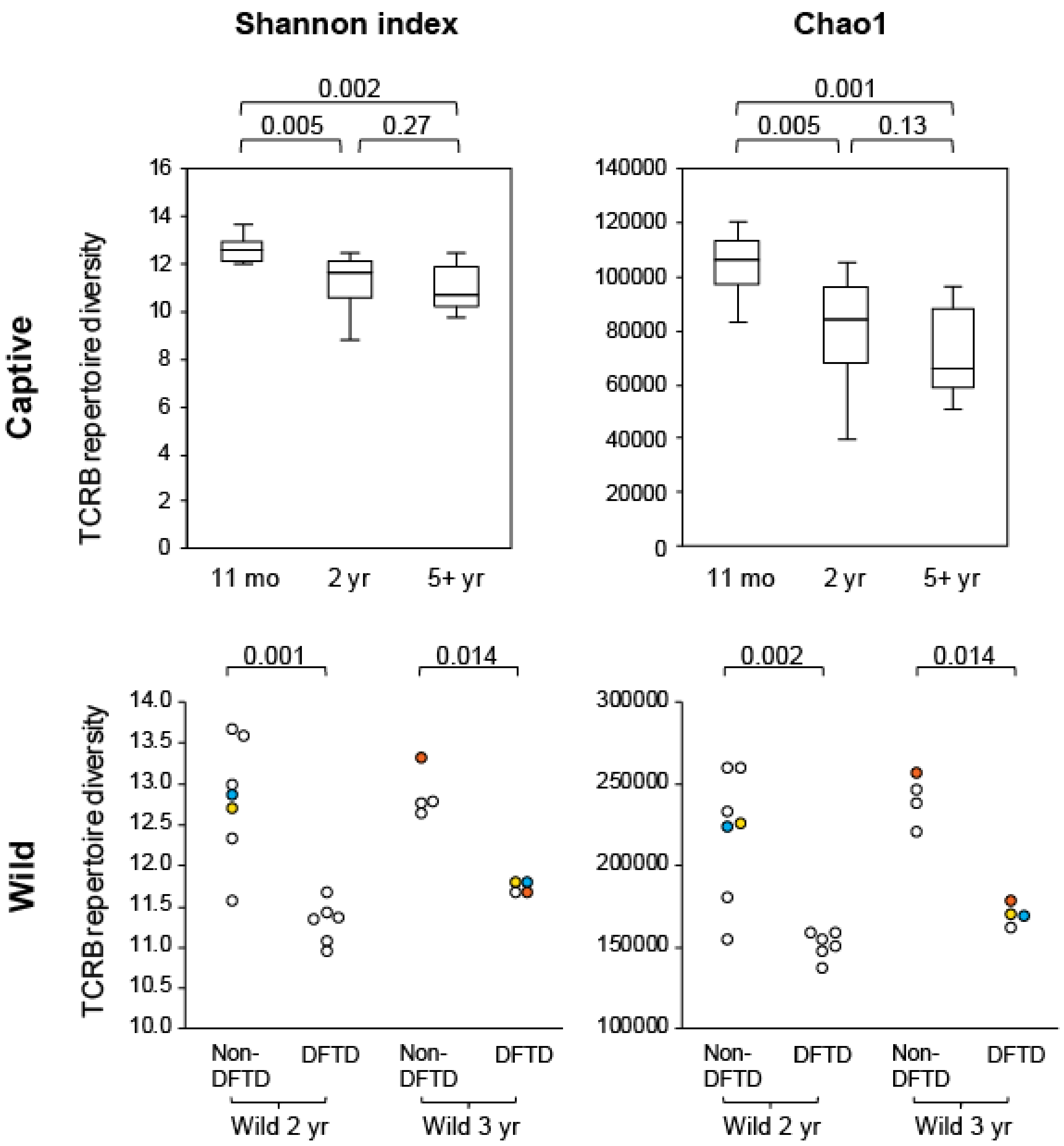

Supplementary Figure 1 TCRB diversity estimated using Shannon index and Chao1 methods. Asterisks indicate p-value < 0.05 using Mann-Whitney U test. Box-plot elements: center line, median; box limits, first and third quartiles; whiskers, minimum and maximum ranges. Coloured data points represent three pairs of pre- and post-DFTD samples, with the same colour indicating the same animal.

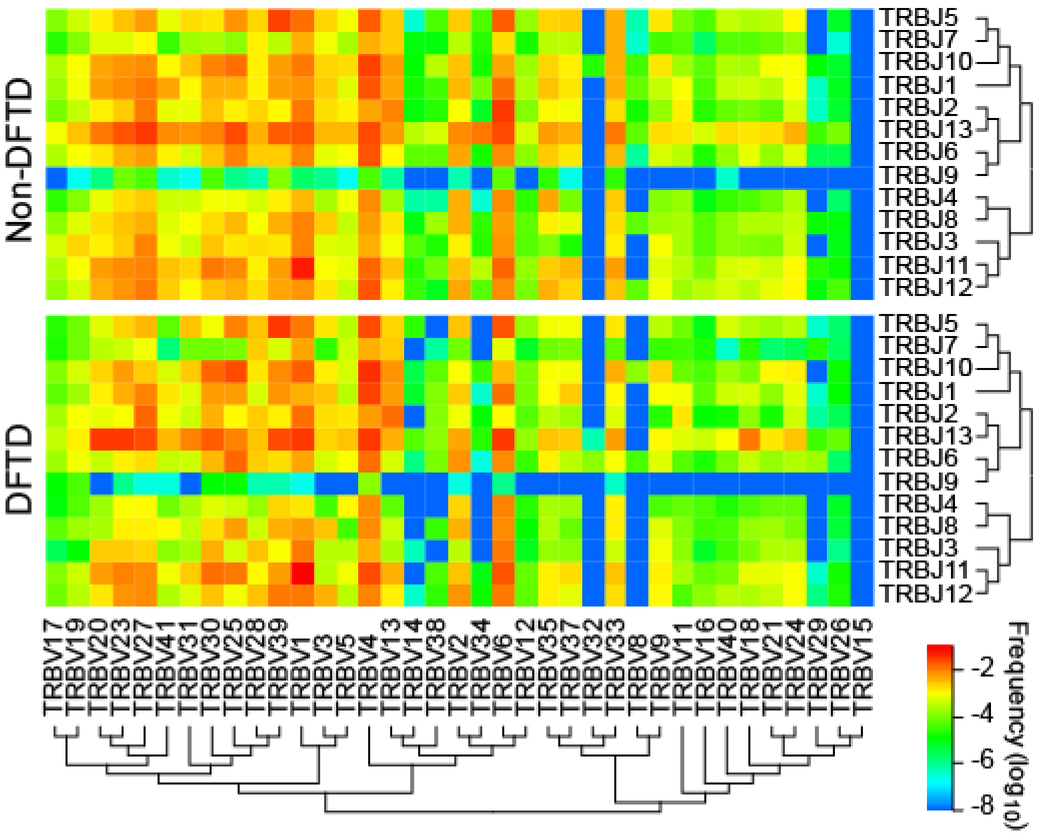

Supplementary Figure 2 The observed frequency of VJ combinations in DFTD and non-DFTD devils. VJ combinations with observed frequency=0 were given a log10 value of -8 (blue squares). V and J segments on x and y axis, respectively, are arranged based on phylogenetic relatedness inferred by Neighbor-Joining method.

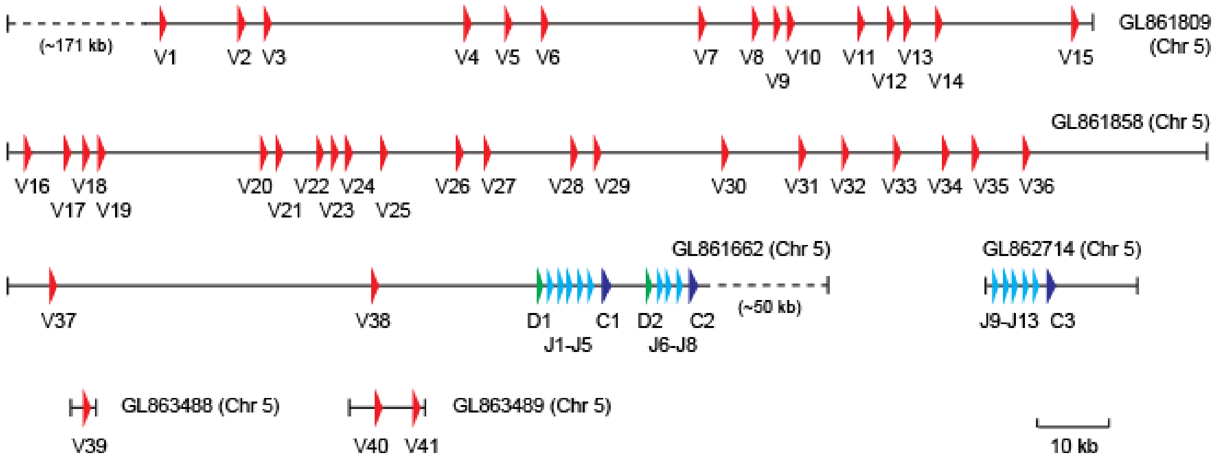

Supplementary Figure 3 Schematic map of devil TCRB locus

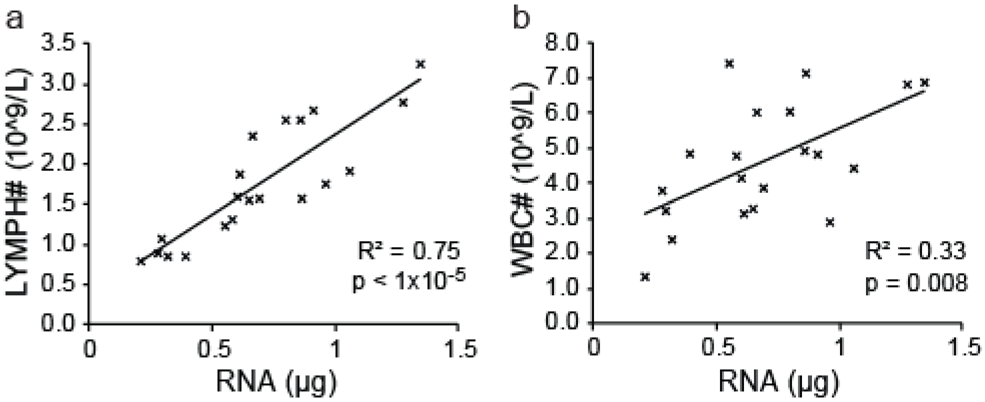

Supplementary Figure 4 Significant correlations between the amount of RNA per 100 µl blood and (a) lymphocyte count, and (b) white blood cell count.

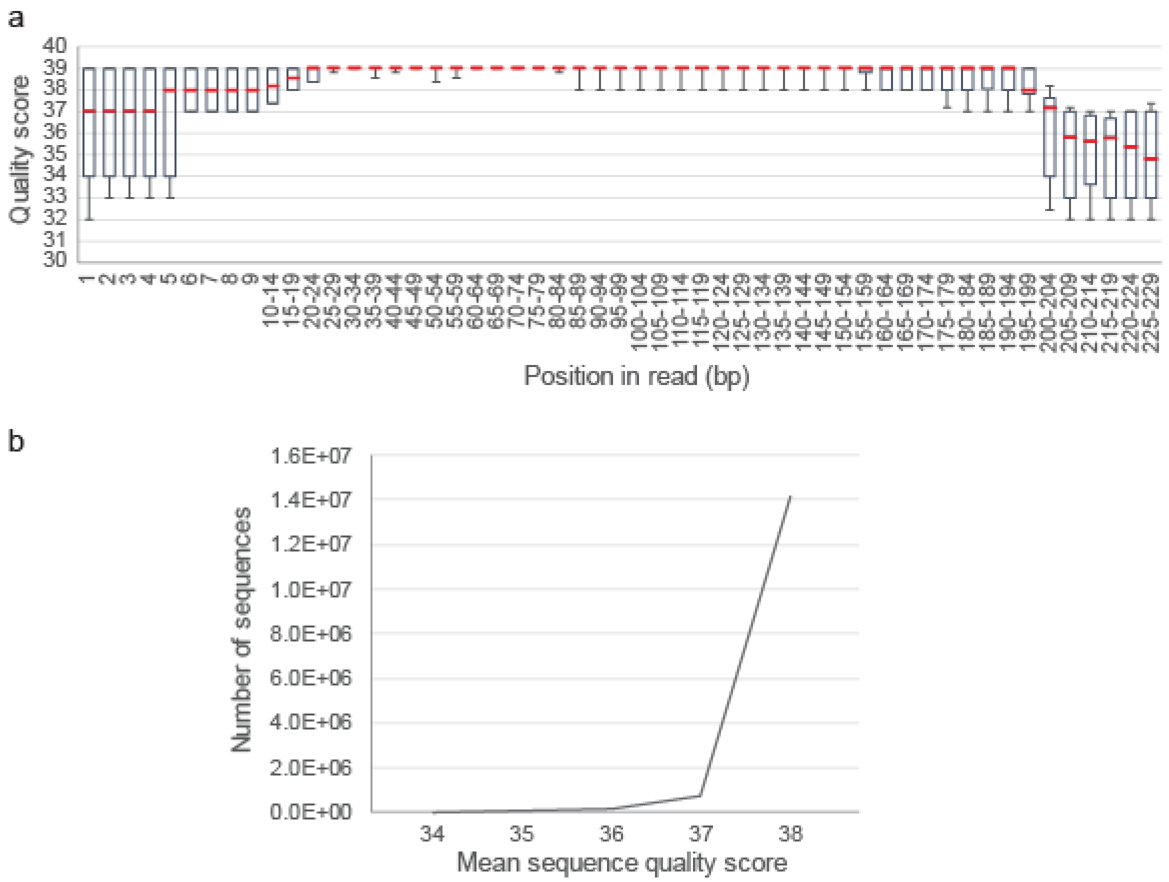

Supplementary Figure 5 Sequence quality control (FastQC). (a) A quality score of 40 indicates 99.99% base call accuracy, and score 30 indicates 99.9% accuracy. (b) Over 98% of sequences have a mean quality score higher than 38.

Supplementary Table 1 Sample information

Population	Sample ID	Age (yr)	DFTD	RNA Integrity Number (RIN)	Number of sequences
Captive	DA33	11 months	N	9.4	169,675
Captive	DA34	11 months	N	9.3	121,502
Captive	DA35	11 months	N	9.4	139,670
Captive	DA36	11 months	N	9.5	88,965
Captive	DA37	11 months	N	9.5	92,537
Captive	DA38	11 months	N	9.5	100,928
Captive	DA39	11 months	N	9.5	126,778
Captive	DA40	11 months	N	9.4	191,526
Captive	DA41	11 months	N	9.1	248,953
Captive	DA42	11 months	N	9.4	185,303
Captive	DA8	2	N	9.2	161,614
Captive	DA9	2	N	9.4	208,319
Captive	DA13	2	N	9.4	126,406
Captive	DA14	2	N	9.5	109,230
Captive	DA15	2	N	9.5	120,794
Captive	DA16	2	N	9	145,954
Captive	DA21	2	N	9.5	151,436
Captive	DA25	2	N	9.3	74,313
Captive	DA27	2	N	9.3	196,313
Captive	DA28	2	N	9.2	314,926
Captive	DA4	5	N	9.2	169,844
Captive	DA11	5	N	9.4	135,984
Captive	DA19	5	N	9.4	124,109
Captive	DA22	5	N	8.7	140,589
Captive	DA24	5	N	9.2	145,902
Captive	DA26	5	N	9.4	64,822
Captive	DA30	5	N	9.5	153,276
Captive	DA31	5	N	9.3	230,729
Captive	DA32	5	N	9.6	162,556
Captive	DA29	6	N	9	236,091
Captive	BJ	7	N	9	225,279
Captive	Oreo	8	N	9.5	230,550
Wild	TD2	2	N	9.5	456628
Wild	TD3	2	N	9.6	446875
Wild	TD5	2	N	9.1	479442
Wild	TD6	2	N	9.7	390821
Wild	TD8	2	N	8.7	464348
Wild	TD1	2	Y	8.5	380051
Wild	TD4	2	Y	9.7	410628
Wild	TD7	2	Y	9	297690
Wild	TD19	2	Y	8.8	293995
Wild	TD20	2	Y	9.3	285501
Wild	TD21	2	Y	8.9	480643
Wild	TD9	3	N	9.4	257416
Wild	TD10	3	N	9	266266
Wild	TD11	3	N	8.5	279657
Wild	TD14	3	Y	8.6	253535
Wild	TD15[a]	2	N	8.5	325668
Wild	TD13[a]	3	Y	9	279796
Wild	TD18[b]	2	N	8.9	412959
Wild	TD17[b]	3	Y	8.8	406465
Wild	TD16[c]	3	N	9	263963
Wild	TD12[c]	4	Y	9.4	290626

[a, b, c] Paired samples, each pair from the same animal before and after catching DFTD.

Supplementary Table 2 Annotation of TCRB gene segments in the Tasmanian devil genome

TRB segment	Scaffold #	Orientation	Coordinates	
V1	GL861809.1	Forward	170853	171147
V2	GL861809.1	Forward	182063	182359
V3	GL861809.1	Forward	185630	185902
V4	GL861809.1	Forward	214310	214601
V5	GL861809.1	Forward	220105	220399
V6	GL861809.1	Forward	225314	225611
V7	GL861809.1	Forward	247609	247885
V8	GL861809.1	Forward	255171	255478
V9	GL861809.1	Forward	258415	258676
V10	GL861809.1	Forward	260288	260548
V11	GL861809.1	Forward	270163	270466
V12	GL861809.1	Forward	274594	274844
V13	GL861809.1	Forward	276893	277204
V14	GL861809.1	Forward	281396	281678
V15	GL861809.1	Forward	300807	301079
V16	GL861858.1	Forward	1422	1720
V17	GL861858.1	Forward	7083	7414
V18	GL861858.1	Forward	9647	9945
V19	GL861858.1	Forward	11763	12054
V20	GL861858.1	Forward	34945	35236
V21	GL861858.1	Forward	37024	37313
V22	GL861858.1	Forward	43104	43398
V23	GL861858.1	Forward	45181	45512
V24	GL861858.1	Forward	46940	47229
V25	GL861858.1	Forward	52193	52415
V26	GL861858.1	Forward	62934	63173
V27	GL861858.1	Forward	66600	66891
V28	GL861858.1	Forward	79076	79370
V29	GL861858.1	Forward	82500	82763
V30	GL861858.1	Forward	100622	100916
V31	GL861858.1	Forward	111551	111845
V32	GL861858.1	Forward	117900	118174
V33	GL861858.1	Forward	125149	125452
V34	GL861858.1	Forward	132010	132337
V35	GL861858.1	Forward	136370	136664
V36	GL861858.1	Forward	143681	143900
V37	GL861662.1	Forward	6142	6434
V38	GL861662.1	Forward	52032	52325
D1	GL861662.1	Forward	79306	79317
J1	GL861662.1	Forward	80095	80141
J2	GL861662.1	Forward	80230	80277
J3	GL861662.1	Forward	81094	81143
J4	GL861662.1	Forward	81816	81864
J5	GL861662.1	Forward	82723	82771
C1	GL861662.1	Forward	84895	86321
D2	GL861662.1	Forward	92717	92733
J6	GL861662.1	Forward	93342	93388
J7	GL861662.1	Forward	93620	93673
J8	GL861662.1	Forward	94005	94056
C2	GL861662.1	Forward	94910	96445
J9	GL862714.1	Reverse	18888	18934
J10	GL862714.1	Reverse	18617	18667
J11	GL862714.1	Reverse	18428	18476
J12	GL862714.1	Reverse	18251	18300
J13	GL862714.1	Reverse	17857	17902
C3	GL862714.1	Reverse	11781	13451
V39	GL863488.1	Reverse	1733	2022
V40	GL863489.1	Forward	3787	4059
V41	GL863489.1	Forward	9875	10162

Supplementary Table 3 TCRB primers

Primer name	Primer sequence (5'-3')	Amplified TRBV	Efficiency[a]
TRBV1	GGAACTTCACTAACAACGATCA	V1	0.96
TRBV11	CAGCTATGAGAAGGAGTTTGAT	V11	1.00
TRBV12	CCAAGCATAGATTCTCAACTGAA	V12	0.96
TRBV13	AGAGTTATTGTTTGCGAAGAGAT	V13	0.98
TRBV14	AGGCAAAGTTATTGTTCTTGAAG	V14	0.97
TRBV15	GATAATATAGAGGATGGTGACTA	V15	0.98
TRBV16	TACTCTSTGGGAAAGAATAACRCAG	V16,V18	1.02
TRBV17	CCTCCGCRATTCAAGCCTAC	V17,V19	0.95
TRBV2	CAGTTCACAGTTGTTCCAGAAT	V2	0.99
TRBV20	CCTCCCAGATTCAAGCCTAC	V20,V23,V27	1.02
TRBV21	TACTCTGTGGGAAAGGATAATACAG	V21,V24,V40	1.00
TRBV25	ACCGTTCCTCCAAGATTCAAAC	V25,V28,V39	1.02
TRBV26	GCTTGTTCTTTACTCAGTATCAAAGG	V26	1.01
TRBV29	TTGTTCTTTATTCTCTGGGAAAGG	V29	0.96
TRBV3	CGCTTTCAATCAGGTTTCCATAA	V3	0.97
TRBV30	GACTGTTCCTCCAAGATTTGAAC	V30	0.98
TRBV31	AATCGATTTGATCCTATATCATCAG	V31	1.02
TRBV32	GGGATATAATGTCATTCGGAAAG	V32	0.99
TRBV33	TCACACGGAAAGAAAAGGAGATA	V33	0.96
TRBV34	GGTGTGCCAAAAACTCGATTC	V34	1.03
TRBV35	TAGACAAAGGAGAAGTCTCAGAT	V35,V37	0.99
TRBV38	GGGTGGATAAGTCTAGGTTTAC	V38	0.97
TRBV4	GATTCACAGCTAAGCAGGATAA	V4	0.98
TRBV41	GAGACTGTTTCTCTGCGATTC	V41	0.98
TRBV5	GCTTCAGCCCAGACTCTTC	V5	0.98
TRBV6	ATGCACAAACCTCGATTCTCA	V6	1.01
TRBV8	TAAATAGCACTCCAGACAACTTC	V8	1.03
TRBV9	CCAAAGAAAAGGACATCTCTGAA	V9	0.97
TRBC_R	AGTCACYTTTGGGGGAGTCA		

[a] Primer efficiencies analysed with real-time PCR using the same standard sample and method as described in Supplementary Methods.

Supplementary Table 4 qPCR primers and efficiency

Gene	Primer (5'-3')	Reaction efficiency	Correlation coefficient of standard curve
GAPDH[1]	F: GACTCAACCACGTATTCGGCTC	0.99	0.999
	R: ATATGATTCCACCCATGGCAAGTTCAA		
GUSB[2]	F: CTGCTGCCTATTATTTCAAGAC	1.02	0.997
	R: CAAGATCCAATTCAGGCTTAG		
IL10[2]	F: GGCAGAGAATGAAACGGAGA	1.00	0.988
	R: ACTTCACAAGGCAGGAATCTGT		
IL6[2]	F: GCACTAAAAATGTTCACGACTC	1.04	0.990
	R: CACTCATTCAGGCTCTTCAG		
TGFB1[2]	F: TATGGACATTAGTGGTGAGTG	1.03	0.990
	R: AAGGACGGTTCTGGTTCT		
TBX21[3]	F: AGCCTCCAATAATGTGACTCAG	1.02	0.993
	R: GTGAAGGTGTGGGTCAAAGAG		
GATA3[3]	F: CACAGGGTTCGGATGTAAGTC	0.97	0.998
	R: CAGTACCATCTCTCCTCCACAA		
IFNG[3]	F: AGTTCTTCTGGCTGTCTTTCTC	1.02	0.991
	R: CCCTCTTTCCAAGTCTTCATCA		
IL4[3]	F: GTCCACGGACAGAGAAGAAACTG	0.97	0.991
	R: ATGTCTAGCACTTCCATCTCAGAG		
FOXP3	F: CCATGCAGGCCCATCTCTCT	0.97	0.991
	R: GGCAGTAGCTGGCTTTCTCTGT		
CTLA4	F: AGCTTCACCATCCCAAGAACTG	0.98	0.995
	R: CAAAGCTGGCAACCCCTCTG		

Supplementary Methods

Annotation of devil TCRB genes

Three TRBC genes were identified by performing BLASTN searches in the devil reference genome using opossum TRBC coding sequences (available at the IMGT database http://www.imgt.org/). Using predicted coding sequences of devil TRBC genes, TBLASTX searches were performed to isolate TCRB transcripts in the devil cDNA database on Ensembl (available at http://www.ensembl.org/Sarcophilus_harrisii/). Twenty-three TCRB cDNA sequences were found, which were mapped to 21 V segments on five genome scaffolds. The recombination signal sequence (RSS) with 23-bp spacer flanking the 3' side of these V segments were extracted and aligned to generate a profile hidden Markov model using software HMMER 3.1b2, and HMM searches were carried out to further identify potential RSS associated with V elements on the TCRB scaffolds. Thirty-two more RSS were found this way, 20 of which had in-frame putative TRBV coding sequences located upstream. Using the above 41 identified V segments, a final batch of whole genome BLASTN search was performed and no further TRBV was found.

Sample collection

Approximately 1 ml blood per kg body mass (no more than 5 ml in total) was sampled from each animal. Blood was collected into RNAprotect Animal Blood 500 µl Tubes (Qiagen) for RNA extraction. For cell counting, blood was collected in EDTA tubes and samples were either examined on the same day of collection or preserved with Streck Cell Preservative™, which qualitatively and quantitatively stabilises leukocyte subsets in the blood, and counted within a week.

PCR protocol for TCRB primers

PCR was carried out using Platinum Taq DNA Polymerase High Fidelity Kit (Invitrogen) in a total volume of 10 µl, which contains 1 µl 10x High Fidelity PCR Buffer, 2 mM $MgSO_4$, 0.2 mM dNTPs, 0.3 µM each primer, 1 U Platinum Taq DNA Polymerase High Fidelity, and approximately 60 ng cDNA. PCR cycling steps included 94 °C for 2 minutes; 32 cycles of 94 °C for 15 seconds, 60 °C for 30 seconds, and 72 °C for 1 minute; and 72 °C for 10 minutes.

Computed tomography

The animals were fasted for 12 hours before anaesthesia was carried out by applying 5% isoflurane through a face mask placed over the nose and mouth. Anesthetized animals were then positioned on the CT table lying on their sternum with 3% isoflurane administered for maintenance of anaesthesia. All CT scans were performed using a 16-slice multi-detector CT scanner (Phillips 16 Slice, Brilliance™ CT V2.3, Phillips Medical Systems Netherlands). Both pre- and post-contrast helical scans of the entire body were acquired. Both animals received a dose of 2 ml per kg of iohexol (350 mg of iodine per mL) (Omnipaque, GE Healthcare Australia Pty Ltd), which was administered intravenously. The tube settings for the CT scan were the same for every scan: beam collimation of 16 data channels with a detector-row width of 0.75 millimeters (16 x 0.75 mm), a tube potential of 120 kVp and a tube current of 150 mA. Images were reconstructed using soft tissue and bone kernels with a slice thickness of 1mm.

Quantitative PCR protocols

Real-time PCRs were carried out on a RotorGene 6000 in a total volume of 20 µl, containing 10 µl 2x Quantifast Sybr Green PCR Master Mix (Qiagen), 0.5 µM each of forward and reverse primers, and approximately 60 ng cDNA. All samples were analysed in triplicates with no-template negative controls included for each gene in each run. PCR conditions were as follows: an initial enzyme activation step of 95 °C 5 minutes; 40 cycles of two-step cycling of 95 °C for 10 seconds and 60 °C for

30 seconds; a final heating step from 50 °C to 99 °C with fluorescence signal collected every 1 °C to generate a melting curve. A composite standard containing equal parts of cDNA from eight wild samples was made and included in each run. To estimate PCR efficiencies, standard curves were generated for all genes by making five 1:3 serial dilutions of the composite standard sample; PCR efficiencies were calculated as -1+10^(-1/slope). The same approach and composite standard sample were used for estimating efficiencies of TCRB primers (Supplementary Table 3).

Supplementary References

1 Murchison, E. P. *et al.* The Tasmanian Devil Transcriptome Reveals Schwann Cell Origins of a Clonally Transmissible Cancer. *Science* **327**, 84-87, doi:10.1126/science.1180616 (2010).

2 Morris, K. & Belov, K. Does the devil facial tumour produce immunosuppressive cytokines as an immune evasion strategy? *Vet Immunol Immunopathol* **153**, 159-164, doi:http://dx.doi.org/10.1016/j.vetimm.2013.02.008 (2013).

3 Cheng, Y. *et al.* Significant decline in anticancer immune capacity during puberty in the Tasmanian devil. *Sci Rep* **7**, 44716, doi:10.1038/srep44716 (2017).
