# Supplementary material for: Tasmanian devils with contagious cancer exhibit a constricted T-cell repertoire diversity
Source: Commun Biol. 2019 Mar 13;2:99. doi: 10.1038/s42003-019-0342-5 (PMC6416256; doi:10.1038/s42003-019-0342-5)
Supplement: Supplementary file 2 — Description of Additional Supplementary Files [file 42003_2019_342_MOESM2_ESM.docx]

**Description of Additional Supplementary Files**

**File Name**: Supplementary Data 1

**Description**: Sequences of top-100 most abundant public clonotypes that showed significantly higher frequencies in DFTD samples than in non-DFTD samples (fasta format)

**File Name**: Supplementary Data 2

**Description**: Observed frequencies of top-100 most abundant public clonotypes that showed significantly higher frequencies in DFTD samples than in non-DFTD samples (tab delimited with header line)
