# Supplementary material for: Tasmanian devils with contagious cancer exhibit a constricted T-cell repertoire diversity
Source: Commun Biol. 2019 Mar 13;2:99. doi: 10.1038/s42003-019-0342-5 (PMC6416256; doi:10.1038/s42003-019-0342-5)
Supplement: Supplementary file 5 — Reporting Summary [file 42003_2019_342_MOESM5_ESM.pdf]

nature research

Corresponding author(s): Katherine Belov

Last updated by author(s): Jan 21, 2019

Reporting Summary

Nature Research wishes to improve the reproducibility of the work that we publish. This form provides structure for consistency and transparency in reporting. For further information on Nature Research policies, see Authors & Referees and the Editorial Policy Checklist.

Statistics

For all statistical analyses, confirm that the following items are present in the figure legend, table legend, main text, or Methods section.

n/a	Confirmed	
☐	☒	The exact sample size (*n*) for each experimental group/condition, given as a discrete number and unit of measurement
☐	☒	A statement on whether measurements were taken from distinct samples or whether the same sample was measured repeatedly
☐	☒	The statistical test(s) used AND whether they are one- or two-sided<br>*Only common tests should be described solely by name; describe more complex techniques in the Methods section.*
☐	☒	A description of all covariates tested
☒	☐	A description of any assumptions or corrections, such as tests of normality and adjustment for multiple comparisons
☐	☒	A full description of the statistical parameters including central tendency (e.g. means) or other basic estimates (e.g. regression coefficient) AND variation (e.g. standard deviation) or associated estimates of uncertainty (e.g. confidence intervals)
☒	☐	For null hypothesis testing, the test statistic (e.g. F, t, r) with confidence intervals, effect sizes, degrees of freedom and P value noted<br>*Give P values as exact values whenever suitable.*
☒	☐	For Bayesian analysis, information on the choice of priors and Markov chain Monte Carlo settings
☒	☐	For hierarchical and complex designs, identification of the appropriate level for tests and full reporting of outcomes
☒	☐	Estimates of effect sizes (e.g. Cohen's *d*, Pearson's *r*), indicating how they were calculated

Our web collection on statistics for biologists contains articles on many of the points above.

Software and code

Policy information about availability of computer code

Data collection	No software was used.
Data analysis	blast/2.3.0, hmmer/3.1b2, FastQC, fastq-join, cdhit, DivE 1.0, OsiriX, Oligo v6.71, REST 2009 v2.0.13

For manuscripts utilizing custom algorithms or software that are central to the research but not yet described in published literature, software must be made available to editors/reviewers. We strongly encourage code deposition in a community repository (e.g. GitHub). See the Nature Research guidelines for submitting code & software for further information.

Data

Policy information about availability of data

All manuscripts must include a data availability statement. This statement should provide the following information, where applicable:

- Accession codes, unique identifiers, or web links for publicly available datasets
- A list of figures that have associated raw data
- A description of any restrictions on data availability

Sequence data that support the findings of this study have been deposited in NCBI Sequence Read Archive (SRA) with the accession code SRP092288.

Field-specific reporting

Please select the one below that is the best fit for your research. If you are not sure, read the appropriate sections before making your selection.

☒ Life sciences ☐ Behavioural & social sciences ☐ Ecological, evolutionary & environmental sciences

For a reference copy of the document with all sections, see nature.com/documents/nr-reporting-summary-flat.pdf

Life sciences study design

All studies must disclose on these points even when the disclosure is negative.

Sample size	The sample size in this study was mainly determined by the number of Tasmanian devils captured during field trips. All blood samples collected from Tasmanian devils belonging to the examined age classes were used for RNA extraction; all samples that produced RNA with RIN>8.5 were used in the analyses.
Data exclusions	No data were excluded from the analyses.
Replication	Multiple biological replicates (N=7-12) were used for each experimental group to enable inter-group comparison, though most individuals were sampled once only and no technical replicate was performed.
Randomization	Samples were allocated into experimental groups based on the age and disease status of the animals, which are the main factors that can affect an animal's TCR repertoire diversity.
Blinding	The investigators were not blinded to group allocation of samples; however, sample IDs used for data analyses were not assigned based on experimental groups (Supplementary Table 1), and consistent analysis methods and parameters were applied to all samples regardless of group allocation.

Reporting for specific materials, systems and methods

We require information from authors about some types of materials, experimental systems and methods used in many studies. Here, indicate whether each material, system or method listed is relevant to your study. If you are not sure if a list item applies to your research, read the appropriate section before selecting a response.

Materials & experimental systems

n/a	Involved in the study	
☒	☐	Antibodies
☒	☐	Eukaryotic cell lines
☒	☐	Palaeontology
☐	☒	Animals and other organisms
☒	☐	Human research participants
☒	☐	Clinical data

Methods

n/a	Involved in the study	
☒	☐	ChIP-seq
☒	☐	Flow cytometry
☒	☐	MRI-based neuroimaging

Animals and other organisms

Policy information about studies involving animals; ARRIVE guidelines recommended for reporting animal research

Laboratory animals	The study did not involve laboratory animals.
Wild animals	The age and disease status of Tasmanian devils used in the study are provided in Supplementary Table 1. Wild devils were trapped during routine population monitoring trips; samples were collected on site following the Blood Collection from Tasmanian Devils SOP. An animal ethics statement has been included in the manuscript.
Field-collected samples	Blood was collected into RNAprotect Animal Blood 500 µl Tubes (Qiagen) for RNA extraction and into EDTA tubes for cell counting. Samples were kept at 4 °C for short-term (a few hours) storage during transport; the RNAprotect samples were stored at -80 °C.
Ethics oversight	We have complied with all relevant ethical regulations and all experiments and procedures that involved the use of Tasmanian devils were carried out with approval from the Animal Ethics Committee of The University of Sydney under project number 550 (captive devils) and 681 (wild devils).

Note that full information on the approval of the study protocol must also be provided in the manuscript.
